# 546. Transmission of Candida auris FKS1 Mutations in Orange County, CA

**DOI:** 10.1093/ofid/ofaf695.019

**Published:** 2026-01-11

**Authors:** Jennifer Brown, Mi Le, Denise Tirol, Elizabeth Kryger, Gabrielle Villareal, Cherry Q Fontela, Angelica Torres, Tania Chiem, Victoria Buchanan, Curtis J Kapsak, Megan Crumpler, Matthew Zahn

**Affiliations:** Orange County Health Care Agency, Santa Ana, California; Orange County Health Care Agency, Santa Ana, California; Orange County Health Care Agency, Santa Ana, California; Orange County Health Care Agency, Santa Ana, California; Orange County Health Care Agency, Santa Ana, California; Orange County Health Care Agency, Santa Ana, California; Orange County Health Care Agency, Public Health Laboratory, Irvine, California; Orange County Health Care Agency, Public Health Laboratory, Irvine, California; Orange County Health Care Agency, Public Health Laboratory, Irvine, California; Theiagen Genomics, Highlands Ranch, Colorado; Orange County Health Care Agency, Public Health Laboratory, Irvine, California; Orange County Health Care Agency, Santa Ana, California

## Abstract

**Background:**

*Candida auris (C. auris)* is a potentially multidrug-resistant yeast that can cause severe infections in high-risk patient care populations and is endemic in Orange County (OC). U.S. isolates remain largely susceptible to echinocandins, the first-line therapy for most invasive infections, though echinocandin resistance (Ech-R) is emerging. FKS1 mutations associated with Ech-R have been thought to be primarily driven by antifungal exposure, though a recent study indicated apparent transmission in U. S. healthcare settings (MMWR). We investigated a cluster of 9 OC *C. auris* cases with FKS1 mutations identified by whole genome sequencing (WGS) analysis.

**Figure:**
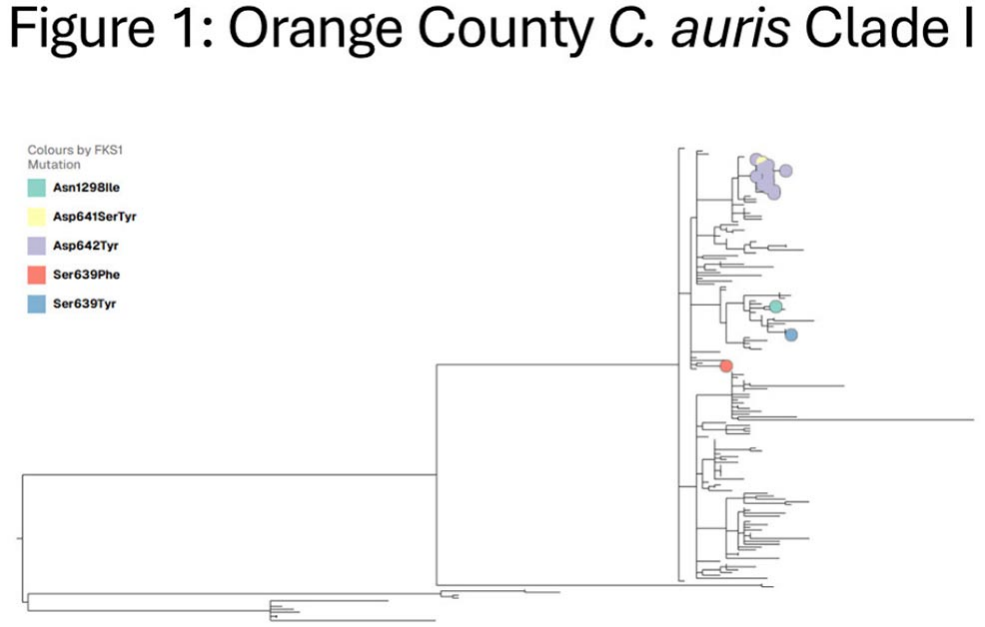

**Figure:**
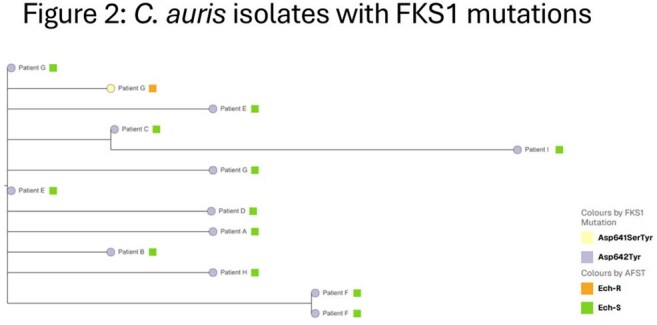

**Methods:**

3,366 OC cases were identified from Feb 2019 - Mar 2025. A total of 601 isolates from 549 cases (16%) underwent WGS at the OC Public Health Lab for clade and FKS1 mutation identification. 988 isolates from 688 cases (20%) underwent Antifungal Susceptibility Testing (AFST) at the Antimicrobial Resistance Laboratory Network (ARLN).

Cluster identification, WGS single nucleotide polymorphism (SNP) analysis and phylogenetic tree development were supported by Theiagen Genomics and Nevada State Public Health Lab.

Epidemiological data including healthcare facility movement, patient exposure, and antifungal treatment was obtained for all cases with FKS1 mutations.

**Figure:**
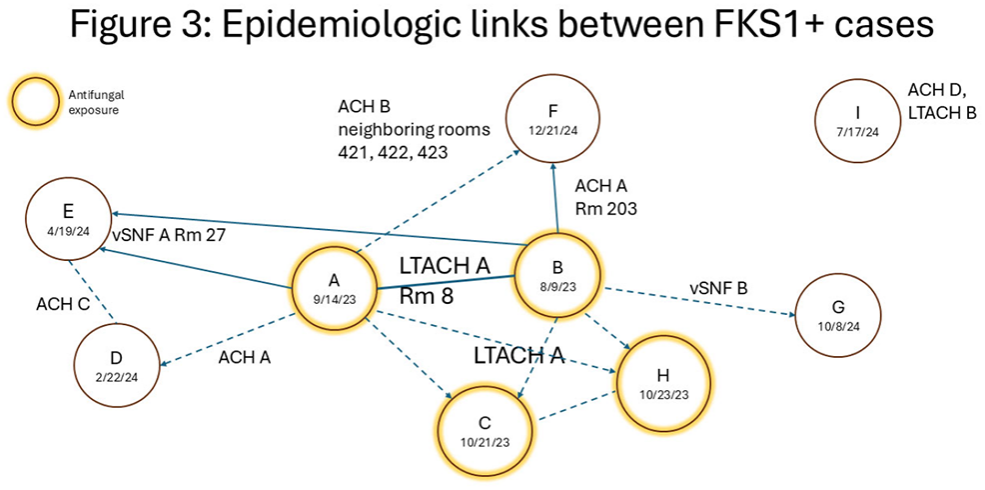

**Results:**

FKS1 mutations were detected in 4 (1%) Clade III and 19 (10%) Clade I isolates. Eight distinct FKS1 mutations were identified. Isolates from 9 cases with a single FKS1 mutation were clustered in the Clade I tree (0-8 SNP differences). The 2 initial cases were roommates at an LTACH. Six of 7 subsequent cases resided in the same facility as one of the initial cases; 2 stayed in a room immediately after an initial case. The 2 initial cases (A and B, Figure 3) received echinocandin treatment prior to testing culture positive. Two of 7 subsequent cases received pre-culture echinocandins.

**Conclusion:**

Our investigation identified apparent transmission of *C. auris* with an FKS1 mutation involving multiple healthcare facilities and highlights the importance of combining public health epidemiologic investigation with WGS analysis. Person-to-person transmission of *C. auris* with potential echinocandin resistance carries significant infection control implications.

**Disclosures:**

All Authors: No reported disclosures

